# Medical school research ranking is associated with gender inequality in MSTP application rates

**DOI:** 10.1186/s12909-018-1306-z

**Published:** 2018-08-06

**Authors:** Caitlin J. Bowen, Calvin J. Kersbergen, Olive Tang, Andrea Cox, Mary Catherine Beach

**Affiliations:** 1 Medical Scientist Training Program, Johns Hopkins University, 1830 East Monument Street, Suite 2-300, Baltimore, MD 21205 USA; 2 Oncology, Medicine, and Medical Scientist Training Program, Johns Hopkins University, 1830 East Monument Street, Suite 2-300, Baltimore, MD 21205 USA; 3 General Internal Medicine and Berman Bioethics Institute, Johns Hopkins University, 2024 East Monument Street, Baltimore, MD 21287 USA

**Keywords:** MSTP, Gender inequality, Medical school applications, MD-PhD, Academic medicine

## Abstract

**Background:**

The number of female trainees in MD and biomedical PhD programs has reached near parity with their male counterparts for several years. However, a gender disparity persists for enrollment in Medical Scientist Research Programs (MSTPs). Several studies suggest women underestimate their abilities compared with male colleagues. If this phenomenon applies, we might expect there to be a gender disparity in applicants to MSTPs, which are typically considered more competitive compared to MD or PhD programs. In this report, we explored this hypothesis by evaluating whether female applicants who do apply to MSTP programs disproportionately apply to lower ranking programs when compared to male applicants.

**Methods:**

For each institution, we identified their 2016 U.S. News and World Report “Best Medical Schools: Research” ranking and examined trends across rankings using linear regression models, such as relationships between the percentage of female applicants and other factors that may influence where applicants apply.

**Results:**

The female applicants who do apply to MSTP programs apply disproportionately to lower ranking programs. Despite this, women seem to have the same success rate for gaining admission to MSTPs, as indicated by matriculation rates across programs, regardless of program rank.

**Conclusions:**

Our findings of gender disparity in applications to high-ranking but not low-ranking programs support prior hypotheses that under-confidence or lack of encouragement may drive this inequality. This analysis highlights the need for further systematic studies of gender differences in MSTP applicants and the relationship to career trajectories in order to improve the gender disparity that exists in academic medicine.

## Background

The number of female trainees in MD and biomedical PhD programs has reached near parity with their male counterparts for several years in the United States [1, 2]. However, a gender disparity remains for enrollment in Medical Scientist Training Programs (MSTPs), in which both MD and PhD degrees are granted through an integrated curriculum. In 2016, women comprised only 38% of the total enrollment in MSTPs in the United States [1]. Previous studies have suggested that fewer women apply to these programs, and indeed, AAMC data demonstrates that only 41% of 2016 applicants to MSTPs were female [1, 3, 4].

Editorial pieces and prior studies have provided a number of possible hypotheses of why fewer women apply to MD-PhD training programs [1, 5]. The reasons offered include challenges with combining the MD-PhD training and furthering a physician-scientist career with family and childbearing, that women feel they have to be better than their male counterparts to be seen as equals, women not being encouraged to become physician-scientists, and a lack of role models for women aspiring to be physician-scientists and academic researchers. However, many of these concerns also arise for women who aspire to be physicians and biomedical scientists through either the MD or PhD pathways, yet they apply to and matriculate at these programs at rates comparable to men.

Several studies in psychology suggest that women underestimate their abilities compared with male colleagues [6–8]. Similarly, in the medical field, studies have shown that female medical students and surgery residents have lower self-evaluations and tend to underestimate their abilities and performance in surgical clerkships and residency evaluations compared to attending evaluations [9, 10]. If this “self-derogatory” phenomenon was operating in the realms of physician scientist career pursuit, we might expect there to be a gender disparity in applicants to MSTPs, which are typically considered to be more competitive and prestigious. In this research report, we explored this hypothesis by evaluating whether female applicants who do apply to MSTP programs disproportionately apply to lower ranking programs when compared to male applicants.

## Methods

We obtained a roster of MSTP institutions during the 2016–2017 application cycle using National Institute of General Medicine Sciences (NIGMS) data, which is updated annually [11]. For each MSTP-funded institution, we identified their 2016 U.S. News and World Report “Best Medical Schools: Research” ranking [12]. The details of this ranking system can be found online [13]. Briefly, U.S. News and World Report used weighted student selectivity admissions statistics (MCAT, GPA and acceptance rate), faculty-student ratio, and measurements of research activity to determine rankings. This medical school ranking system was selected because it is publicly available and frequently accessed by applicants. For applicants to medical school and MSTP programs, the annual rankings likely play a significant role in determining where to apply – both as a marker of perceived prestige as well as of competitiveness. Studies have shown that US News Rankings impact choice of undergraduate and law schools, where moving up one rank corresponds to reduced acceptance rates, higher matriculation rates, and higher standardized test scores of accepted students [14, 15]. While these rankings may not adequately quantify school quality, they likely influence school choice and perception of prestige for MSTP program applicants in a similar manner to undergraduate and law school applicants.

Using publicly available Association of American Medical Colleges (AAMC) data, we accessed the number of male and female applicants and matriculants to each MSTP-funded institution’s MD-PhD program in the 2016–2017 application cycle (*n* = 19,774 applications) [3]. Additionally, we determined the total MSTP program size and percent of female trainees in each ranked program, again with available AAMC data. We collated the number of male and female applicants and matriculants to each MSTP-funded institution’s MD-only program for an exploratory comparison (*n* = 315,920 applications to the same 46 institutions). With this dataset, we do not have a measurement of an individual’s qualification for an MSTP program, such as standardized test scores, GPAs, or research success. Therefore, the matriculation rate to individual programs was used as a proxy of the quality of applications submitted.

We used scatter plots to visualize the data and linear regression to examine trends across the U.S. News Rankings and to observe relationships between the percentage of female applicants and other factors that may influence where applicants apply. We tested gender-by-U.S. News Rank interaction terms to determine whether U.S. News Ranking had a stronger association with applications from male compared to female applicants. Next, we tested if the number of female students in a given program or female matriculants in 2016 had an association with U.S. News ranking, as a surrogate for applicant success rate. Finally, we tested for an association between the percentage of current female students and the rate of female applicants to the various programs. Linear regression models between genders were compared using an interaction term in the regression model. All analyses were conducted using Stata version 14.0 (StataCorp. 2015. Stata Statistical Software: Release 14. College Station, TX: StataCorp LP).

## Results

**Fig. 1:**
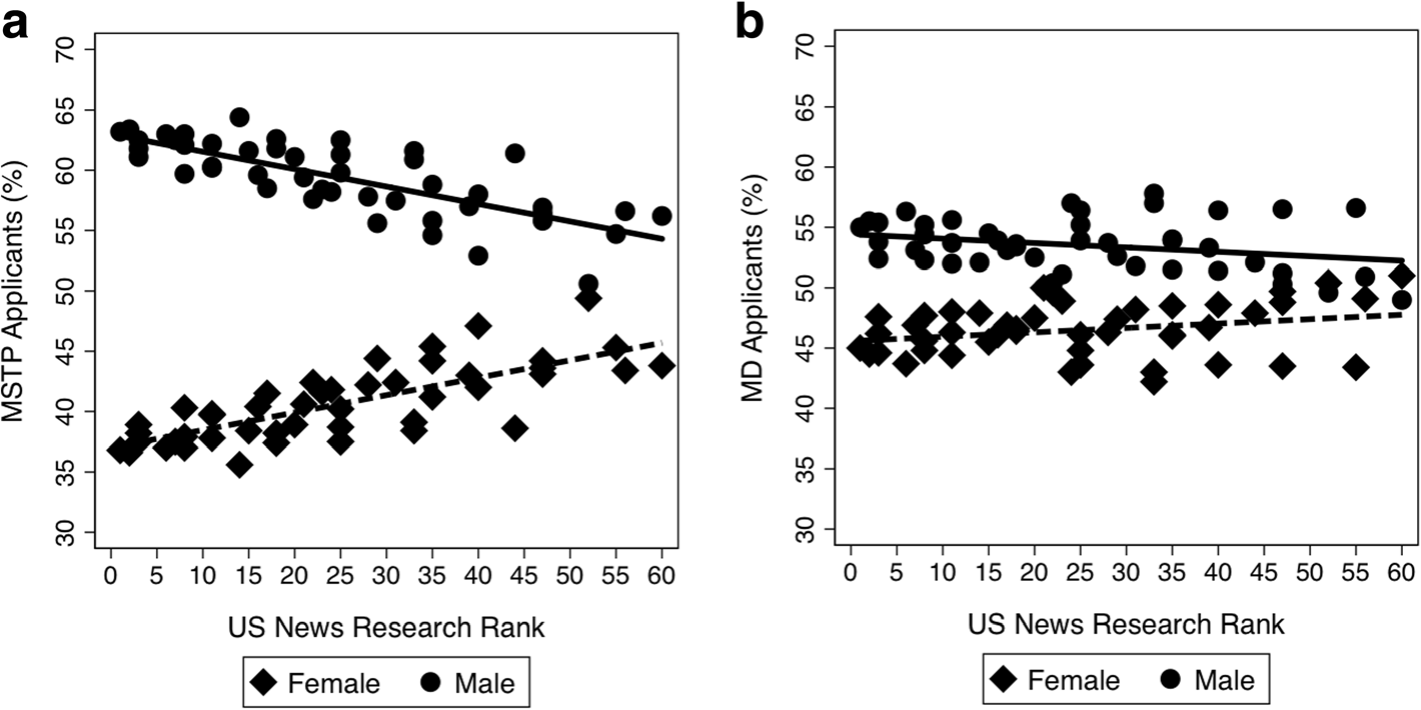
Discrepancies in applicant rates by gender to MSTP (**a**) and MD (**b**) programs based on US News Research Rank. **a** The difference between percent male and female MSTP applicants was greater as the school rank became more competitive (*p*_*interaction*_ *<* 0.001). At schools with a lower US News ranking, the application discrepancy was less pronounced. **b** This trend was not observed among MD applicants to the same schools (*p*_*interaction*_ = 0.621)

**Fig. 2:**
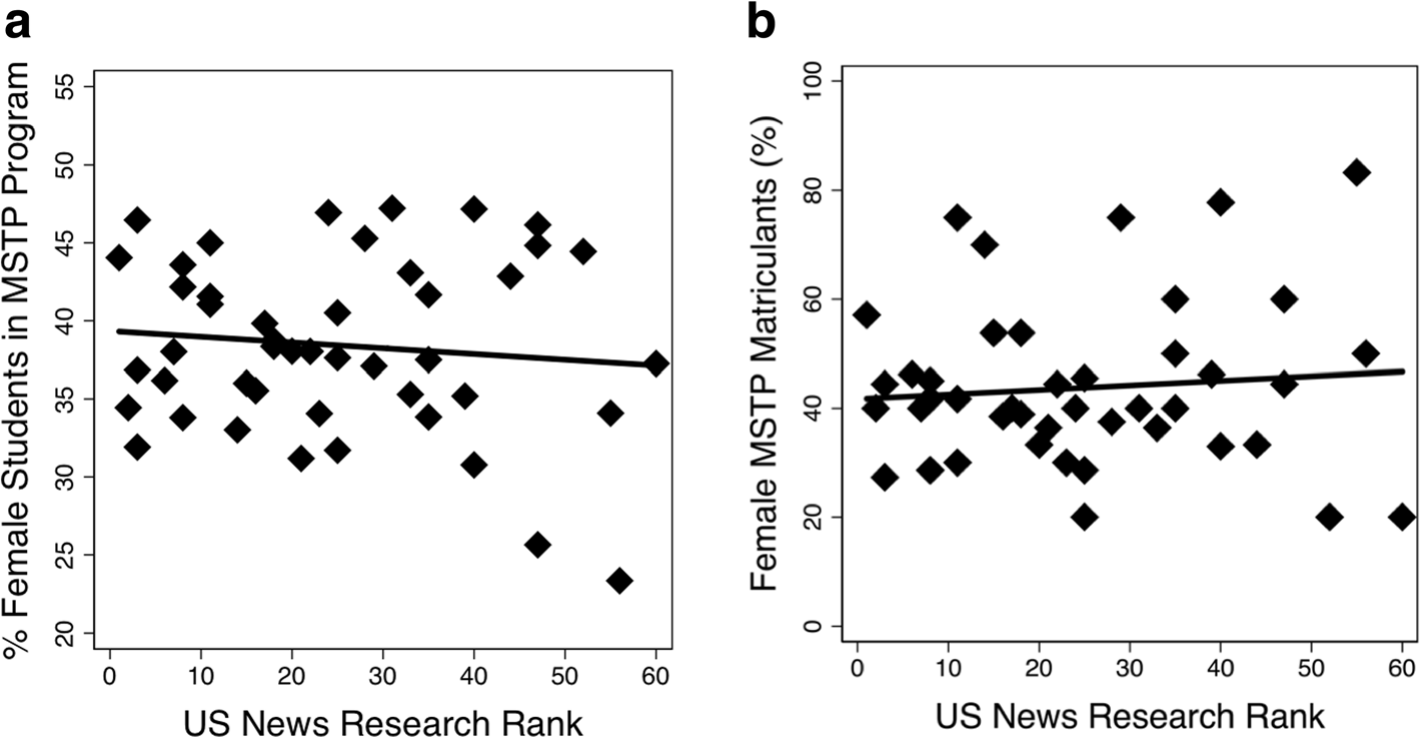
No correlation was observed between percent female students in MSTPs and the US News Research Rank of the program (R^2^ = 0.008; *p* = 0.55) **a** There was also no significant association observed between the percentage of matriculants who were female and the US News Research Rank of the program (R^2^ = 0.0008; *p* = 0.55). One ranked school was excluded because it had no MSTP matriculants in 2016

**Fig. 3:**
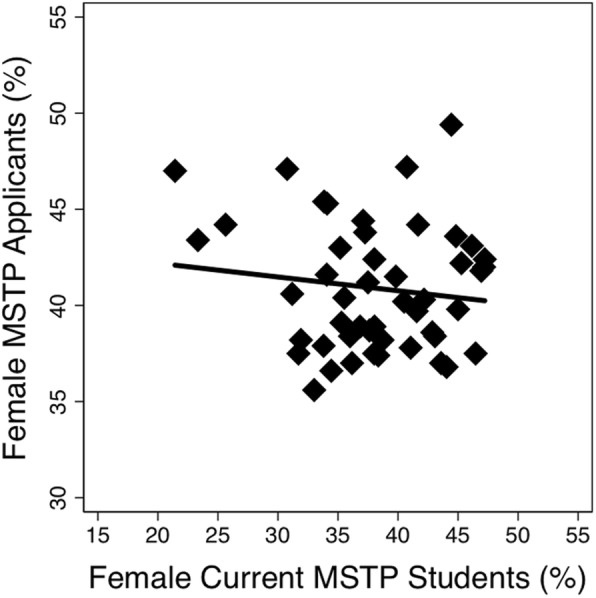
There is no observed relationship between the percent of female MSTP applicants and the percent of female MSTP students in a program (R^2^ = 0.017; *p* = 0.37)

Of 48 MSTP-funded institutions, 46 were ranked by U.S. News and World Report in 2016. Across these 46 MSTP programs, there was a higher percentage of male vs. female applications for both MSTP (59.1 vs. 40.9, *p* < 0.001) and MD (53.5 vs. 46.5, *p* < 0.001) programs. However, the difference between percent male and female MSTP applications was greater as the school rank became more competitive (Fig. 1a; *p*_*interaction*_ *<* 0.001). This trend was not observed in applicants to MD programs at the same medical schools (Fig. 1b; *p*_*interaction*_ *=* 0.621). Despite gender differences in applications based on program rank, we did not observe a correlation between the percent female matriculants to a given MSTP with U.S. News ranking (Fig. 2a; *p* = 0.55) and this seems to hold true over time, as there was no correlation between the percent female students in a given MSTP and the U.S. News ranking (Fig. 2b; *p* = 0.55). Further, there was no correlation between the percent of female students applying to a program and the number of female students enrolled in that program (Fig. 3; *p* = 0.37).

## Discussion

In this study of publicly accessible AAMC data, we demonstrate that the female applicants who do apply to MSTP programs apply disproportionately to lower ranking programs than their male counterparts, a discrepancy not seen with applications to the corresponding MD programs. Despite this discrepancy in application numbers, women seem to have the same success rate for matriculation to MSTPs regardless of U.S. News rank, suggesting that admissions committees consider female applicants as qualified at higher institutions and that this is not driven by bias against them in the admissions process. Further, other factors that may influence where female students apply, such as the number of female students in the program, do not correlate with the number of applications from female students a program receives.

Editorial pieces have suggested reasons why fewer women overall apply to MD-PhD training programs, including challenges with combining the physician-scientist career with family and childbearing, women feeling they have to “super compete”, and a lack of encouragement and/or role models for women [1]. This study builds upon these ideas, suggesting that the challenges facing women who aspire to be physician-scientists may occur early in their career trajectory. This data suggests that the challenges and roadblocks to gender equality are not necessarily inherent to the MSTP program such as program length and combining a future career with family and childbearing, but rather lay in the idea that women may feel they have to be better than their male counterparts to be seen as equals. Therefore, our findings of a gender disparity in applications to high-ranking programs are supportive of the hypotheses that suggest under-confidence and/or lack of encouragement among women aspiring to be physician scientists, relative to men, may be driving this inequality. This hypothesis is supported by psychological literature that suggests that women under-estimate and under-promote their abilities, in particular on tasks, skills, or even careers that are considered masculine [6–8]. These studies have been shown to hold true for female medical students and surgical residents [9, 10]. For female MSTP applicants, a combination of lower self-confidence than males and fewer female physician-scientist role models (giving a perception of physician-scientists as a significantly male-dominated field) may contribute to proportionately fewer women applying to more competitive training programs. With proportionately fewer women applying to more “prestigious” programs, female applicants may be limiting future career opportunities and not be reaching their full potential. Additional studies are needed to further elucidate the underlying reasons for this gender discrepancy.

There was no correlation between either the proportion of MSTP female matriculants in 2016 or the percent of total female MSTP trainees and program ranking, suggesting that this discrepancy isn’t due to a general trend in applications being stronger for males at top-ranked programs. We cannot support this hypothesis explicitly without individual program applicant data from the AAMC, however from our own institution we know that on average male and female applicants have comparable GPA and MCAT scores (personal communication). Publicly available data for all MD programs in 2016 demonstrates that MCAT scores and GPAs were comparable between men and women (503.4 vs 500.4 and 3.55 vs 3.55, respectively) [3]. These data are not publicly available from the AAMC for MSTP programs, but MSTP program applicants are likely as qualified as those applying to MD programs. Interestingly, there was also no correlation between the percent of female applicants and the percent of female students, suggesting that women enroll at similar rates across many programs despite differences in the proportion of applications from female candidates based on program rank and prestige. This could suggest that women may have, in general, stronger applications than men at highly ranking schools, that a gender balance may be a component in some program’s admission processes, or the proportion of women enrolled in the specific program is not a top priority when women are choosing where to apply.

The limitations of this study include reliance on limited data. Without applicant statistics and admissions rates broken down by each school, we can’t assess the association of grades, MCAT scores, research experiences, and other indicators of applicant success and quality in MSTP programs. Additionally, there may be confounders in program size, length of establishment, geographical considerations, or total number of applicants to each program that may influence the schools to which female applicants apply. We only look at the 46 MSTP-funded institutions, which does not include all MD or MD-PhD programs. Further, although MSTP programs are the most straightforward pathway to careers in academic medicine, they are not the only pathway. Women entering physician-scientist careers via alternate routes are difficult to identify, and alternative pathways to an academic medicine career were not included in this analysis.

Additional research is needed to examine the role of gender in association with MSTP program graduates’ career paths. Nearly all (95%) of MD-PhDs who graduated enter residencies and most (81%) are employed in academia, research institutes, or industry [16, 17]. Graduates of MSTPs make up 2.5% of medical school graduates, but account for a third of all NIH research grants awarded to physicians, disproportionately making up more of the active academic medical research community [18]. Therefore, this gender disparity in applications to and enrollment in programs designed to train students for careers as physician-scientists highlights the concerning statistics in female faculty members at U.S. medical schools, which have been described as the “leaky pipeline” for women pursuing careers in academic medicine [19]. Additionally, previous work indicates that there is a progressive loss of female grant applicants and a lower percentage of female than male grant applicants who are successful in obtaining some (e.g., K01 and R01-equivalent), but not all, types of awards [19, 20]. Further research is warranted to fill the gap in knowledge about the physician–scientist career paths of female program graduates.

## Conclusions

Our findings of gender disparity in applications to high-ranking but not low-ranking programs support the prior hypotheses that female applicants may be less confident or lack encouragement, relative to their male counterparts. This in turn may be driving inequality in MSTP applicants and those pursuing academic medicine career paths. This analysis highlights the need for further systematic studies of gender differences in MSTP applicants and experiences and the relationship to career trajectories in order to improve the gender disparity that exists in academic medicine.

## Abbreviations

MSTP: Medical Scientist Training Program
NIGMS: National Institute of General Medicine Sciences
